# Multiparametric MRI of Epiphyseal Cartilage Necrosis (Osteochondrosis) with Histological Validation in a Goat Model

**DOI:** 10.1371/journal.pone.0140400

**Published:** 2015-10-16

**Authors:** Luning Wang, Mikko J. Nissi, Ferenc Tóth, Jonah Shaver, Casey P. Johnson, Jinjin Zhang, Michael Garwood, Cathy S. Carlson, Jutta M. Ellermann

**Affiliations:** 1 Center for Magnetic Resonance Research, Department of Radiology, University of Minnesota, Minneapolis, MN, United States of America; 2 Department of Orthopaedic Surgery, University of Minnesota, Minneapolis, MN, United States of America; 3 Research Group of Medical Imaging, Physics and Technology, Faculty of Medicine, University of Oulu, Oulu, Finland; 4 Medical Research Center Oulu, Oulu University Hospital and University of Oulu, Oulu, Finland; 5 Department of Applied Physics, University of Eastern Finland, Kuopio, Finland; 6 Department of Veterinary Population Medicine, College of Veterinary Medicine, University of Minnesota, St. Paul, MN, United States of America; 7 Laboratory of Nanostructures and Biosensing, Department of Electrical and Computer Engineering, University of Minnesota, Minneapolis, MN, United States of America; University of Pennsylvania, UNITED STATES

## Abstract

**Purpose:**

To evaluate multiple MRI parameters in a surgical model of osteochondrosis (OC) in goats.

**Methods:**

Focal ischemic lesions of two different sizes were induced in the epiphyseal cartilage of the medial femoral condyles of goats at 4 days of age by surgical transection of cartilage canal blood vessels. Goats were euthanized and specimens harvested 3, 4, 5, 6, 9 and 10 weeks post-op. *Ex vivo* MRI scans were conducted at 9.4 Tesla for mapping the T_1_, T_2_, T_1ρ_, adiabatic T_1ρ_ and T_RAFF_ relaxation times of articular cartilage, unaffected epiphyseal cartilage, and epiphyseal cartilage within the area of the induced lesion. After MRI scans, safranin O staining was conducted to validate areas of ischemic necrosis induced in the medial femoral condyles of six goats, and to allow comparison of MRI findings with the semi-quantitative proteoglycan assessment in corresponding safranin O-stained histological sections.

**Results:**

All relaxation time constants differentiated normal epiphyseal cartilage from lesions of ischemic cartilage necrosis, and the histological staining results confirmed the proteoglycan (PG) loss in the areas of ischemia. In the scanned specimens, all of the measured relaxation time constants were higher in the articular than in the normal epiphyseal cartilage, consistently allowing differentiation between these two tissues.

**Conclusions:**

Multiparametric MRI provided a sensitive approach to discriminate between necrotic and viable epiphyseal cartilage and between articular and epiphyseal cartilage, which may be useful for diagnosing and monitoring OC lesions and, potentially, for assessing effectiveness of treatment interventions.

## Introduction

Juvenile Osteochondritis Dissecans (OCD) is a developmental orthopaedic disease affecting children and young adults that results in chondro-osseous flap formation in diarthrodial joints and can lead to significant disability later in life [1]. Unfortunately, OCD usually is diagnosed late in its course, after clinical signs of joint pain and locking are present [2]. Recently, it has been highlighted that no significant progress had been made in understanding the etiology of OCD in humans since its discovery over a hundred years ago [3]. Etiological factors under consideration include genetic [2], traumatic [4] and vascular causes [5]. OCD is highly prevalent in horses and pigs, in which the preclinical stages of the disease (known as osteochondrosis, OC) are well characterized as areas of necrotic epiphyseal cartilage that occur secondary to damage to cartilage canal blood supply [6]. Noninvasive monitoring of the disease in animals has been limited to demonstrating a delay in endochondral ossification (radio-graphically or by CT), which only becomes apparent after the ossification front reaches the area(s) of necrotic cartilage. A noninvasive method to identify the area of necrosis earlier in the development of the disease, while it is confined to the epiphyseal cartilage, has been lacking. These earliest lesions are characterized by marked changes in the cartilage matrix [7], including a progressive loss of proteoglycans (PG) [8,9] and disorganization of collagen fibrils [7,10], Thus, it is reasonable to expect that MRI methods can be developed for their detection.

It is well established that noninvasive parametric T_2_ [11–13] and T_1ρ_ mapping [14,15] of articular cartilage can detect biochemical alterations in this tissue [16,17]. In articular cartilage it has been shown that MRI T_2_ relaxation times reflect the orientation of the collagen network [11–13,18–20], the collagen content [12,21] and also collagen-associated water [12,13]. T_1ρ_ has been shown to reflect the status of macromolecules in animal models of OA in general [22] and is used for detection of early osteoarthritis (OA) in humans [15,23–25]. Recent studies on models of OA have used adiabatic T_1ρ_ relaxation time and relaxation time along a fictitious field (T_RAFF_) to evaluate the cartilage matrix [26–31]. Adiabatic T_1ρ_ contrast is generated using a train of adiabatic full passage (AFP) pulses of the hyperbolic secant family (HSn, n = 1, 4) that are discriminated from the traditional T_1ρ_ intrinsically. The T_RAFF_ pulse sequence [26,27,30] utilizes amplitude-modulated (AM) and frequency-modulated (FM) pulses operating in the sub-adiabatic regime and generating a fictitious field, about which the spins precess in the 2^nd^ order-rotating frame. Thus, T_RAFF_ is intrinsically different from T_1ρ_, which describes relaxation of spins in the 1^st^ order-rotating frame. By satisfying the adiabatic condition, adiabatic T_1ρ_ is less sensitive to magnetic field inhomogeneity than T_1ρ_ [28,32]. When compared to T_1ρ_ measurements, T_RAFF_ can be performed with reduced radiofrequency (RF) power because the stationary spin-locking field is produced by AM and FM (sine and cosine, respectively) modulated pulses operating in a sub-adiabatic regime [27,30,31]. The RAFF sequence uses less energy in the preparation pulses compared to T_1ρ_, and this can dramatically reduce the specific absorption rate (SAR) [27,30,31]. These advantages are particularly relevant for future clinical applications.

In our previous feasibility study, we developed an animal model of surgically induced OC (ischemic necrosis of epiphyseal cartilage) and described characteristic changes in the cartilage matrix utilizing adiabatic T_1ρ_ [33]. The present study evaluates and compares various parametric MRI methods for the assessment of articular cartilage, unaffected epiphyseal cartilage, and OC lesions in epiphyseal cartilage, and validates the MRI results against those acquired using semi-quantitative histology techniques. We hypothesize that noninvasive parametric MRI is sensitive to the biological changes occurring in epiphyseal cartilage during ischemic necrosis. To test our hypothesis, the T_1_, T_2_, T_1ρ_, adiabatic T_1ρ_ and T_RAFF_ relaxation times were measured and compared with histological findings in a goat OC model in which areas of epiphyseal cartilage necrosis were surgically induced by transection of cartilage canal blood vessels. In addition, relaxation times were evaluated in morphologically normal articular and epiphyseal cartilage.

## Materials and Methods

This study consists of four sequential steps: (1) surgical induction of lesions and sample harvesting, (2) multiparametric MRI, (3) histology, and (4) optical density experiment.

### Step 1: Surgical Induction of Lesions and Sample Harvesting

The surgical procedure has been described previously in detail [33]. To create an area of ischemic necrosis in the epiphyseal cartilage, six 4-day-old goats underwent a surgical procedure to interrupt the cartilage canal vascular supply to a focal area of the central (axial) aspect of the right medial femoral condyle (MFC) [33]. Briefly, goats were anesthetized by with a combination of midazolam and ketamine administered intravenously and anesthesia was maintained by inhalation of sevoflurane vaporized in oxygen. The stifle joint was approached using a parapatellar arthrotomy, and the patella was luxated to expose the axial aspects of the femoral condyles and the intercondylar groove. A custom- made 5 × 5 mm blade or a 3 × 4 mm beaver blade was used to create an incision extending into the epiphyseal cartilage of the MFC from the intercondylar groove parallel with the articular surface [33]. A small lesion (3 × 4 mm^2^ incision) was created in half of the goats and a large lesion (5 × 5 mm^2^ incision) was created in the remaining goats. For 72hours post operatively, goats received flunixin meglumine 1.1 mg/kg SC twice a day and ceftioufur 2.2 mg/kg SC twice a day. Goats with large incisions were euthanized at 3, 5, and 9 weeks after surgery and goats with small incisions were euthanized at 4, 6 and 10 weeks after surgery. The euthanasia was performed by intravenous administration of 100 mg/kg Pentobarbital. The operated distal femora were harvested and MRI experiments were done immediately, after which the specimens were placed in 10% neutral buffered formalin for histological processing. The institutional animal care and use committee of the University of Minnesota approved the animal procedures.

### Step 2: Multi-Parametric MRI

Individual distal femora were suspended in flexible latex containers filled with perfluoropolyether oil for a clean and susceptibility matched background. MRI experiments were conducted using a 9.4 T Varian scanner for small animal studies (Agilent Technologies, Santa Clara, CA, USA). The specimens were placed at the center of a shielded quadrature volume coil (Millipede, Varian NMR Systems, Palo Alto, CA). The coil was used for both RF transmission and signal acquisition. B_0_ field shimming and RF pulse calibration were conducted for the scans of all the samples. All scans were conducted at room temperature (20°C). A 2D fast spin echo (FSE) sequence was used as a readout sequence with five different magnetization preparations to measure T_1_, T_2_, T_1ρ_, adiabatic T_1ρ_ and T_RAFF_ relaxation times, sequentially. Details of the magnetization preparations are summarized in Table 1. Specifically, the parameters of the FSE sequence were TE = 5.38 ms, TR = 5 s, echo train length (ETL) = 8, matrix = 256^2^, one coronal slice = 1 mm, acquisition bandwidth (BW) = 131.6 kHz. The fields-of-view (FOVs) were adjusted between 4 × 4 cm^2^ to 4.6 × 4.6 cm^2^ to adapt to the altering sizes of the specimens, providing a resolution between 156 to 180 μm. Depending on the magnetization preparations, the acquisition time to measure one relaxation constant was about 15 to 20 minutes, resulting in an approximate scan time of two hours for one sample.

**Table 1:** Protocols for MR relaxometry.

Relaxation constant	Protocols
T_1_	Inversion recovery, TI = 0.07, 0.08, 0.1, 0.16, 0.32, 0.64, 1.28, 2.56, 5.12 s
T_2_	Double spin echo preparation, TE = 4, 20, 40, 60, 80, and 100 ms
T_1ρ_	Rect. pulse for spin lock, duration = 0, 10, 20, 40, and 80 ms, γB_1_^max^ = 500 Hz,
Adiabatic T_1ρ_	Train of 0, 4, 8, 12, 16 AFP (HS4) pulses, duration = 6 ms, γB_1_^max^ = 2.5 kHz
T_RAFF_	Train of 0, 8, 16, 24, 32 RAFF pulse, duration = 4.53 ms, γB_1_^max^ = 625 Hz

The binary files containing the MRI raw data in k-space were read into MATLAB (MATLAB R2012b, MathWorks, Natick, MA, USA), and the 2D fast Fourier transform function provided in MATLAB was then used to reconstruct the MRI images. T_1_ was calculated using pixel-by-pixel fitting to the equation $Signal(TI)=A\cdot (1-2e^{-TI/T_{1}})$. In the equations, A is a scaling factor, TI is given in Table 1. Other relaxation times were calculated using pixel-by-pixel fitting to the equation *Signal*(*t*) = *A* ⋅ *e* ^−*t*/*T*^. Here, T denotes for corresponding relaxation constants and t represents TE for T_2_ mapping, or spin lock duration for T_1ρ_ mapping (durations of the rect pulses), or pulse duration multiplied by the number of pulses for adiabatic T_1ρ_ and T_RAFF_ mapping (values presented in Table 1). The regions-of-interest (ROIs) of the articular cartilage and the viable and necrotic epiphyseal cartilage were determined by evaluating all types of the MR parametric images. All ROIs were manually selected utilizing Aedes software (http://aedes.uef.fi).

### Step 3: Histology

Following the MR scans, the distal femoral specimens were fixed in 10% neutral buffered formalin for 48 hrs, then decalcified by immersion in 10% ethylenediaminetetraacetic acid. After decalcification, the femoral condyles were serially sectioned in the coronal plane matching the MRI studies into 2 to 3 mm thick slabs, which were routinely processed into paraffin blocks. Sections of 5μm thickness were obtained from the surface of each block and stained with hematoxylin and eosin (H&E). If a lesion of cartilage necrosis was present in the H&E-stained sections, additional sections were obtained at 200 to 500 μm intervals deep to the original block face until the lesion was no longer apparent, to ensure that its entire extent was sampled. Selected sections containing areas of chondronecrosis (characterized by the presence of chondrocytes with pyknotic nuclei and by decreased staining of the extracellular matrix) were also stained with safranin O in order to qualitatively evaluate the loss of PG content. The area of OC lesions was measured using SPOT^TM^ imaging software.

### Step 4: Optical Density Experiment

Representative safranin O-stained sections obtained from animals at different time points after surgery were characterized by transmission imaging microscopy at the light absorption peak of the dye (530 nm) [26]. Regions including normal vs. necrotic epiphyseal cartilage were illuminated by a broad band, laser driven light source (EQ-99FC, Energetiq, MA, USA) filtered with a 15 nm band-pass filter centered at 530 nm at a low magnification on a TI-S microscope (Eclipse Ti-S, Nikon, Japan) coupled to a high resolution, deep cooled EMCCD (iXon Ultra 897, Andor, UK). Reference transmission images were collected from a sample-free area of the glass slide. In order to compensate for differences in exposure time required to avoid camera saturation, sample and reference image counts were normalized to counts per second after background subtraction. Sample images were divided by reference images to get transmittance images, the negative log of which provided the optical density indicative of the light absorption in the tissue. The intensity of safranin O staining is known to be directly related to the optical density and, correspondingly, to the proteoglycan content of cartilage [34–36].

To illustrate the predictive capability of the parametric MRI, the percent difference of the light absorption between the viable and necrotic epiphyseal cartilage was scatter-plotted with the percent difference of the measured relaxation constants between the viable and necrotic epiphyseal cartilage. A regression analysis was also conducted by fitting the data linearly to estimate the relationship between the proteoglycan content and the MRI relaxation constants.

## Results

### Histological Characteristics of the Necrotic Epiphyseal Cartilage

**Fig 1:**
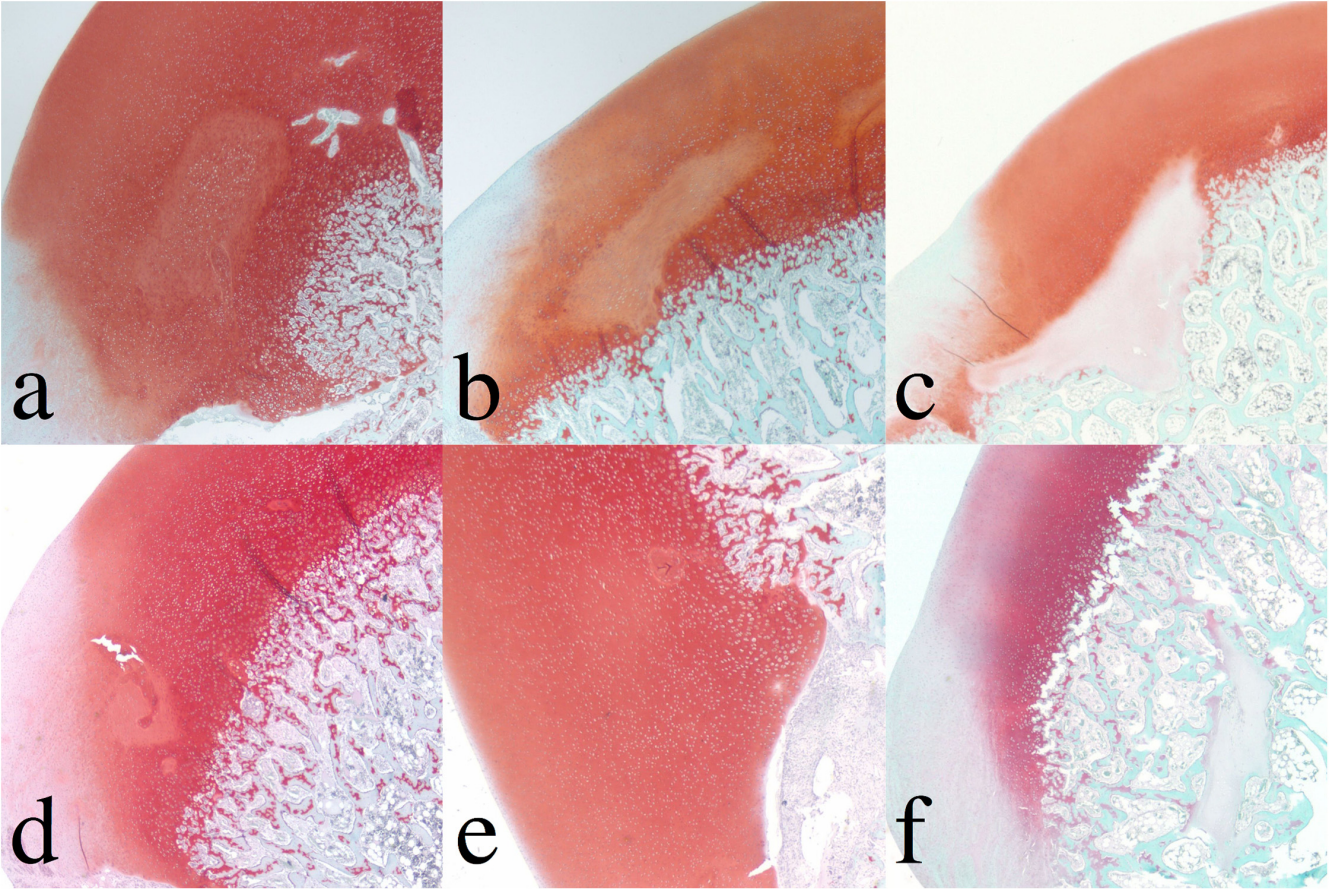
Safranin O-stained sections of femoral condyle. Top row: Surgically induced large lesions (3, 5, and 9 weeks post induction). Bottom row: Surgically induced small lesions (4, 6 and 10 weeks post surgical induction). Decreased staining in the chondronecrosis shows a variable degree of pallor. The optical density experiment of the safranin O-stained sections of the femoral condyle was conducted and shown in Fig 2 to estimate the PG loss in the chondronecrosis.

In 5 out of 6 goats, surgical interruption of the vascular supply to the MFC resulted in a well-circumscribed area of epiphyseal cartilage necrosis, characterized by necrotic chondrocytes within an area of matrix that exhibited a variable degree of pallor in the safranin O stained (Fig 1) and H&E-stained sections [33]. The lesion in the goat that was euthanized 6 weeks after surgery contained no histologically identifiable area of cartilage necrosis (Fig 1E). The average size of the area of cartilage necrosis in the goats in which a small (3 × 4 mm^2^) incision was made was 0.55 mm^2^ (range 0.5–0.6 mm^2^) whereas that in which the larger (5 × 5 mm^2^) incision was made was 2.8 mm^2^ (range 1.2–5.3 mm^2^) [33]. None of the lesions involved the overlying articular cartilage, which is avascular throughout life and does not depend on a blood supply from cartilage canal vessels.

**Fig 2:**
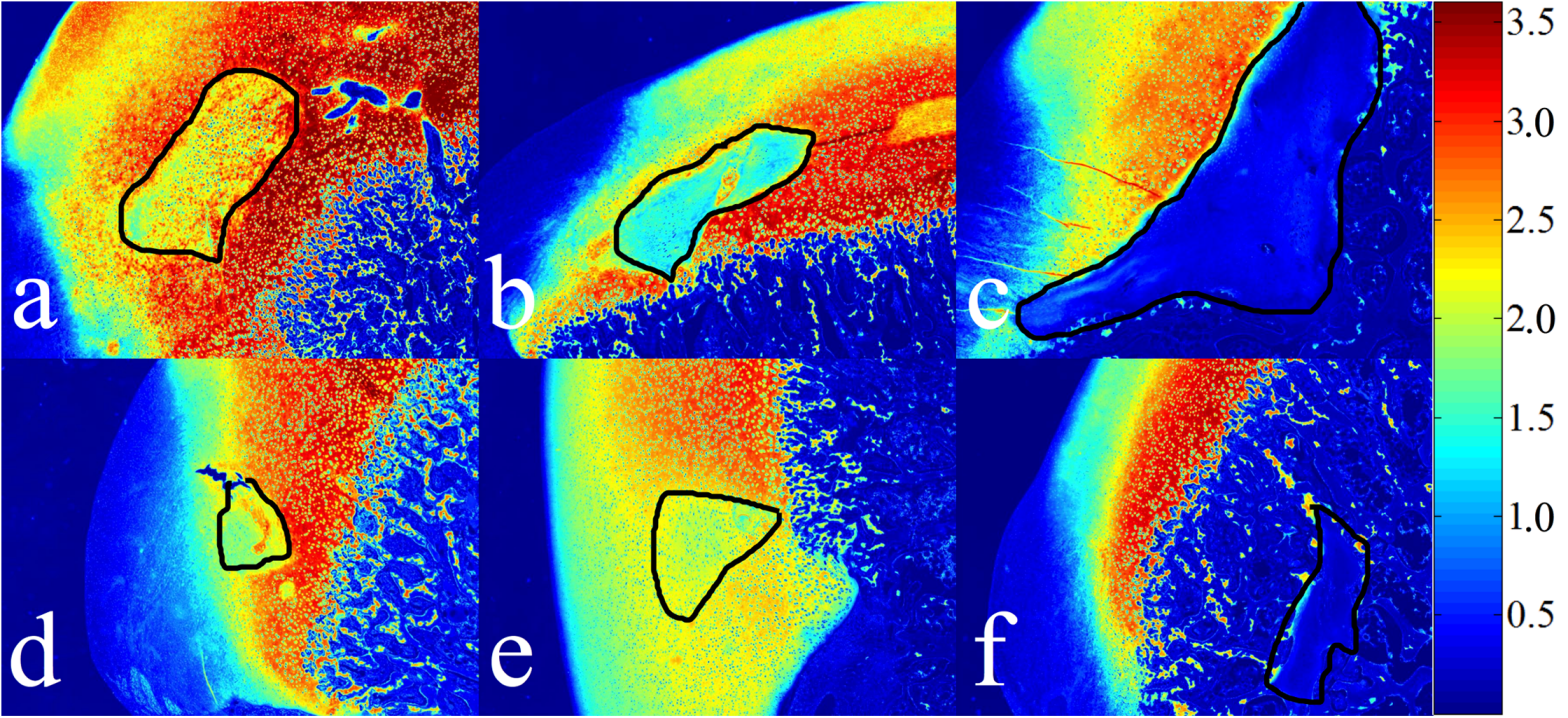
Light absorption (arbitrary units [A.U.]) in safranin O stained sections of femoral condyle. Top row: Surgically induced large lesions (3, 5, and 9 weeks post induction). Bottom row: Surgically induced small lesions (4, 6 and 10 weeks post surgical induction). The areas of chondronecrosis are outlined in black (the area in (E) is selected based on the PG loss, in which the color shows yellow or light blue). Intralesional color spectrum ranges from yellow to dark blue, as proteoglycans are progressively lost from the cartilage matrix. The late-stage lesions in (C) and (F) similarly demonstrated very low proteoglycan content and either resulted in a marked delay in endochondral ossification (C) or became completely surrounded by bone (F).

**Fig 3:**
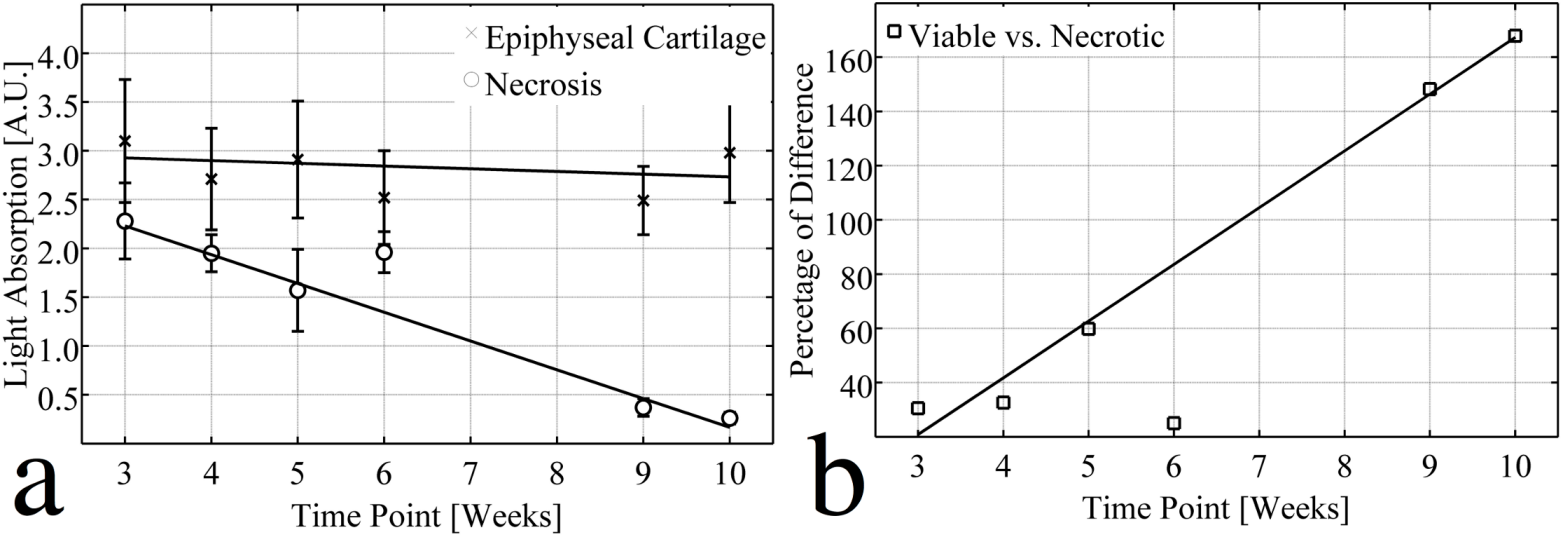
Linear regression analysis of the optical density experiment. (A) Light absorptions (A.U.) for viable (x, R^2^ = 0.129) and necrotic (o, R^2^ = 0.989) epiphyseal cartilage were fitted linearly to different time points, indicating that the PG content remained stable in the viable epiphyseal cartilage, but decreased linearly with time in the areas of chondronecrosis. (B) The % difference between light absorption by viable and necrotic epiphyseal cartilage was fitted linearly to different time points (R^2^ = 0.993). Although slight PG loss was observed in the 6-week sample, this point was excluded from the analysis because no histologically evident chondronecrosis was observed.

Decreased safranin O staining associated with PG loss resulted in a diminished light absorption in areas of cartilage necrosis (inside the black curves in Fig 2). Although no cartilage necrosis was identified in the sample obtained 6 weeks after surgery (Fig 1E), light absorption was decreased in the area adjacent to the incision (Fig 2E). The numerical values of the relative light absorption were established for the necrotic cartilage (inside all enclosed curves in Fig 1) and the normal epiphyseal cartilage (outside the curves in Fig 1, excluding articular cartilage). The optical density of viable epiphyseal cartilage fluctuated in a narrow range between 2.49 and 3.10 in all samples (Table 2) and indicated that the PG content was closely similar in the normal epiphyseal cartilage among the different juvenile goats. In contrast, light absorption decreased in areas of necrotic cartilage with increasing time after surgery, from 2.28 three weeks after surgery to 0.26 ten weeks after surgery (Table 2). The difference between light absorption of viable vs. necrotic cartilage increased linearly over time from 30.5% three weeks after surgery to 168% ten weeks after surgery (Fig 3). The lesion without histologically identifiable necrosis (6 weeks post surgery), however, only had 25% less light absorption than the normal epiphyseal cartilage and appears as an outlier when the data are graphed (Table 2 and Fig 3).

**Table 2:** Light absorption (optical density) of the safranin O-stained epiphyseal cartilage.

Time [weeks]	Viable	Necrotic	Difference
3	3.10±0.63	2.28±0.39	30.5%
4	2.71±0.52	1.95±0.19	32.6%
5	2.91±0.60	1.57±0.42	59.8%
6	2.52±0.48	1.96±0.21	25.0%
9	2.49±0.35	0.37±0.09	148.2%
10	2.98±0.51	0.26±0.06	167.9%

### Multiparametric MRI of the Epiphyseal Cartilage

In all scanned samples, all of the measured relaxation constants were higher in the articular than in the viable epiphyseal cartilage and also were higher in the lesions than in the viable epiphyseal cartilage (Table 3). The percentage increase between viable epiphyseal cartilage and the lesions varied among the different relaxation parameters, reflecting different sensitivities to the induced necrosis (Table 4). The comparison between goats that received large and small incisions indicated that both increased size and duration of the induced necrosis caused relaxation times to increase (Table 4). Unlike the progressive PG loss observed histologically, the MRI relaxation times did not change linearly with the time after surgery (Tables 3 and 4).

**Table 3:** MRI relaxation constants of the articular and sub-articular viable and necrotic epiphyseal cartilage.

Time [weeks]	Cartilage	T_1_ [s]	T_2_ [ms]	T_1ρ_ [ms]	Adiabati T_1ρ_ [ms]	T_RAFF_ [ms]
3	Articular	1.56±0.14	-	133.7±27.0	275.8±51.3	248.7±49.0
	Viable	1.23±0.06	-	90.8±7.4	193.3±14.5	133.2±12.3
	Necrotic	1.61±0.08	-	137.9±12.6	288.8±24.1	244.0±21.0
5	Articular	1.51±0.04	92.5±8.4	123.7±12.4	252.0±29.1	149.0±9.9
	Viable	1.17±0.03	54.6±4.4	88.0±3.9	180.2±8.8	102.4±4.6
	Necrotic	1.91±0.09	106.6±13.1	205.3±18.1	413.3±42.1	230.2±22.4
9	Articular	1.45±0.07	74.2±7.4	99.2±9.1	188.9±17.6	143.0±11.5
	Viable	1.31±0.06	59.6±9.5	89.1±7.4	180.7±14.7	127.0±11.3
	Necrotic	2.08±0.08	136.4±17.9	207.3±18.5	414.3±61.6	286.4±34.5
4	Articular	1.62±0.16	68.4±15.9	95.2±24.2	223.0±48.3	175.7±35.9
	Viable	1.34±0.06	32.6±8.7	57.2±9.1	157.9±16.2	100.8±14.3
	Necrotic	1.38±0.04	44.0±3.4	64.4±3.5	162.5±10.4	126.4±5.1
6	Articular	1.78±0.24	84.8±16.9	100.1±21.6	283.2±61.0	144.1±28.4
	Viable	1.29±0.04	22.8±5.9	58.2±4.3	147.9±9.9	60.7±9.6
	Necrotic	1.45±0.05	46.1±4.8	79.7±3.7	181.7±11.2	100.1±7.1
10	Articular	1.58±0.13	61.9±10.0	85.7±13.9	201.6±37.0	134.4±19.4
	Viable	1.27±0.04	23.0±5.9	53.5±4.2	134.7±9.6	69.0±6.6
	Necrotic	1.56±0.17	38.9±18.4	128.5±19.5	234.2±35.4	137.3±11.2

Large lesion (5 × 5 mm incision): 3, 5, 9, weeks post-surgery

Small lesion (3 × 4 mm incision): 4, 6, 10 weeks post-surgery

Due to technical problems, the T_2_ measurement was invalid for the 3-week specimen and was not included.

**Table 4:** Percent difference in relaxation times between viable and necrotic epiphyseal cartilage using various MRI sequences.

Time [weeks]	T_1_	T2	T_1ρ_	Adiabatic T_1ρ_	T_RAFF_
3	26.9%	-	41.2%	39.6%	58.7%
5	47.9%	64.5%	80.0%	78.6%	76.8%
9	45.2%	78.4%	79.8%	78.5%	77.1%
4	3.3%	29.7%	11.8%	2.8%	22.5%
6	11.3%	67.8%	31.2%	20.5%	49.1%
10	20.9%	51.6%	82.5%	54.0%	66.3%

Large lesion (5 × 5 mm incision): 3, 5, 9, weeks post surgery

Small lesion (3 × 4 mm incision): 4, 6, 10 weeks post surgery

Due to technical problems, the T_2_ measurement was invalid for the 3-week specimen and was not included.

**Fig 4:**
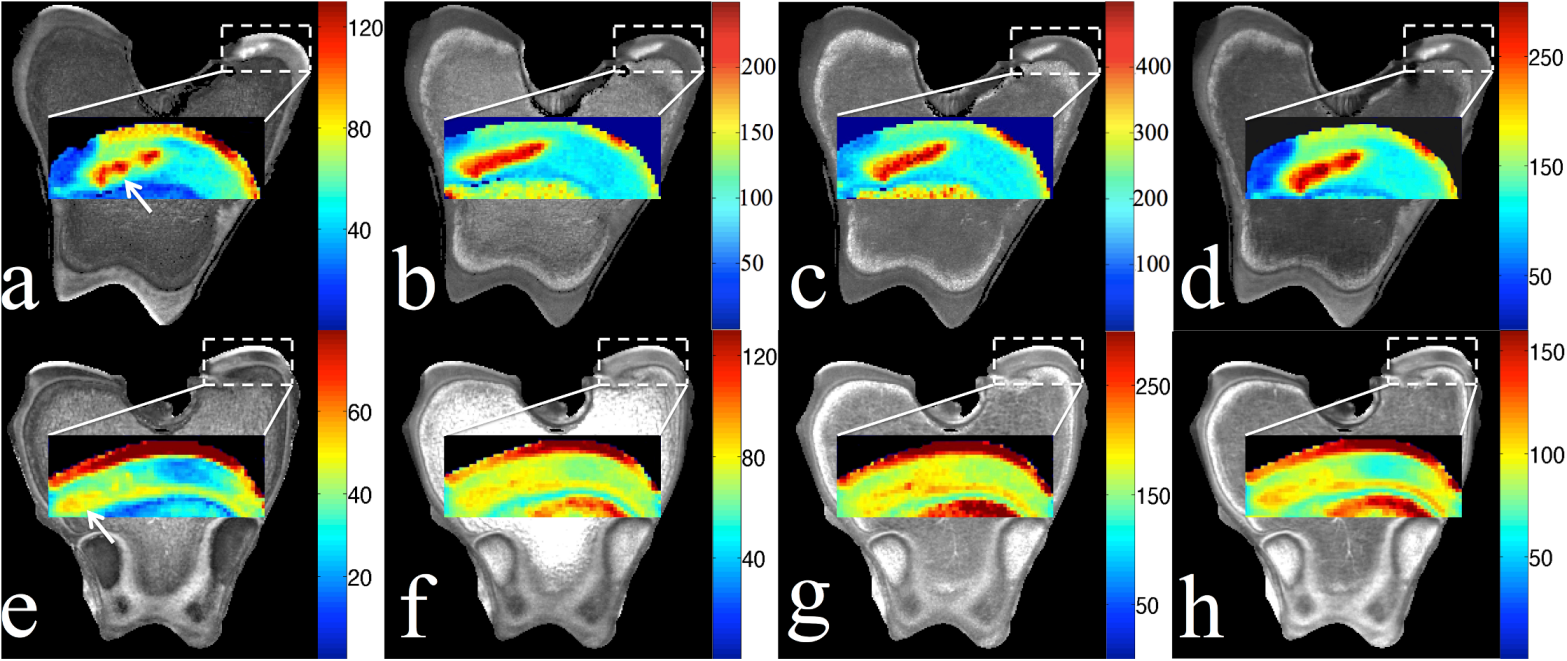
Parametric MRI images of femoral condyle. Relaxation time maps for (A, E) T_2_, (B, F) T_1ρ_, (C, G) adiabatic T_1ρ_, and (D, H) T_RAFF_ in the medial condyle of the distal femur at 5 weeks (top row, large lesion) and 6 weeks (bottom row, small lesion) post-surgically. Lesion locations are indicated by the arrows in the T_2_ maps.

Two representative cases, one with a small incision (6 weeks post surgery) and one with a larger incision (5 weeks post surgery) were selected to illustrate the parametric MRI images (Fig 4). T_2_, T_1ρ_, adiabatic T_1ρ_, and T_RAFF_ values were increased in the lesions compared to normal epiphyseal cartilage in both cases. T_1_ was not included in the figure due to its limited sensitivity to the induced necrosis. With the large incision, up to an 80% increase in the relaxation times made it easier to discriminate the necrotic from viable epiphyseal cartilage in the images (Table 4 and Fig 4). With the small incision, the relaxation times increased less, but the lesion could still be identified in the images, even though cartilage necrosis was not identified histologically and the PG loss was very limited (Tables 2 and 4 and Fig 4).

Interestingly, T_RAFF_ increased 58.7% and 22.5%, in the 4-week (small lesion) and the 3-week (large lesion) samples, although the PG loss was similar in these cases (Tables 2 & 4). At the early time points (3 and 4 weeks post surgery), T_2_ and T_RAFF_ appeared to be more sensitive to the cartilage necrosis than other sequences, evidenced by the higher average difference between these two relaxation constants for necrotic and viable cartilage compared to those for T_1ρ_ and adiabatic T_1ρ_ (Table 4). Conversely, at intermediate and later time points (5, 9, 10 weeks post surgery) where PG loss became severe (Table 2), T_1ρ_ and adiabatic T_1ρ_ appeared to be more sensitive to detecting cartilage necrosis compared to T_2_ and T_RAFF_ (Table 4). In the specimen harvested 6 weeks after surgery, where no histologically apparent necrosis was present and the percentage of PG loss was the lowest at 25% (Table 2), marked differences for T_2_ (67.5%) and T_RAFF_ (49.1%) were observed between the lesion and the normal surrounding epiphyseal cartilage. The respective changes for T_1ρ_ (31.2%) and adiabatic T_1ρ_ (20.5%) were smaller (Table 4).

**Fig 5:**
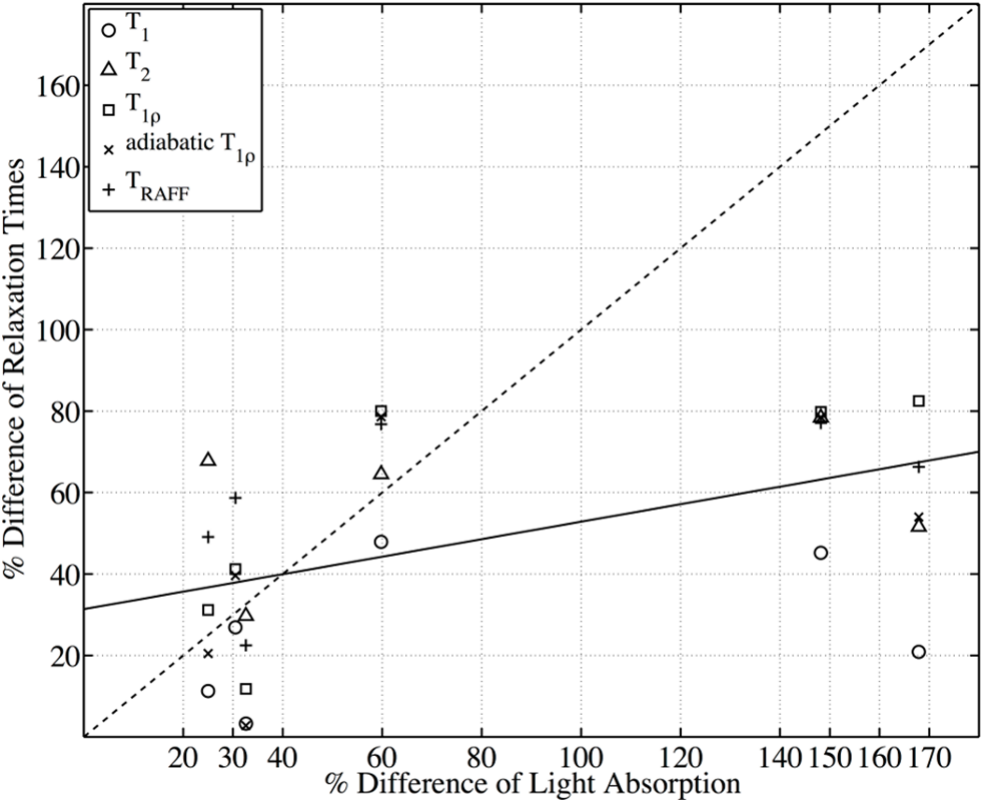
Regression analysis of the percent differences of all parametric MRI relaxation constants and of the light absorption between the viable and necrotic epiphyseal cartilage. The solid line shows the linear fitting (R^2^ = 0.39) between the percent differences of all MR relaxation constants (Table 4) and the percent difference of the light absorption (Table 2, column 4), resulting in the slope equal to 0.21.

The regression analysis in Fig 5 compares the percent difference of the light absorption (fourth column in Table 2) vs. the percent difference of the measured relaxation constants (Table 4) between the viable and necrotic epiphyseal cartilage. The measured percent difference of relaxation constants did not change proportionally with that of the light absorption, which is proportional to the PG loss. The slope of the fitted line (solid, R^2^ = 0.39) is about 0.21, indicating that the relaxation time constants generally had less percent change than the corresponding light absorption. When the difference in light absorption is less than 60%, T_2_, T_1ρ_, adiabatic T_1ρ_, and T_RAFF_ generally appear above the dashed line (slope = 1), meaning these relaxation constants were more sensitive than the optical density experiment at the early stage of the disease. However, when PG was dramatically lost (the percent difference of the light absorption >140%), it can be seen that the relaxation constants reached a plateau and did not increase further.

## Discussion

These results demonstrate that multiple established and novel parametric MR methods allow noninvasive detection of chondronecrosis, one of the earliest changes associated with OC, in the epiphyseal growth cartilage. Both classical parametric mapping techniques that are available on all clinical scanners and novel techniques allowed identification of early and late stages of chondronecrosis associated with surgically induced small and large OC lesions. The noninvasive character of these MRI techniques will facilitate the investigation of OCD in humans and are likely to shed light on the etiology and pathophysiology of the disease.

While there is an abundance of literature about articular cartilage parametric mapping using conventional [11,12,15], or novel techniques [26,28], these noninvasive methods have not previously been applied and compared for the evaluation of disorders involving the sub-articular epiphyseal growth cartilage. Although the major components of hyaline cartilage matrix, proteoglycans, collagen, and water are found in both articular and epiphyseal cartilage, there are fundamental differences between these two tissues, as evidenced by tissue extraction methods [8,37]. For example, it has been shown that highly active remodeling with selective resorption of Type II collagen takes place in epiphyseal cartilage while proteoglycan content is found to be relatively stable [37]. Ischemic cartilage necrosis, occurring secondary to failure of cartilage canal blood supply in OC, is associated with degradation of collagen into smaller fibrils and loss of organization [38].

Our results also verified that T_2_, T_1ρ_ and, additionally, adiabatic T_1ρ_ and T_RAFF_ are able to detect areas of cartilage necrosis in both early and late stages. We noted, however, that the tested relaxation times (Table 3 and Fig 5) are not proportional to PG content in cartilage, or to the duration of the lesions. In the regression analysis, they appear sensitive to PG content at the early stage of the disease at a time when the PG loss is not dramatic (less than 60%), but reached a plateau in the late stage. It is likely that multiple factors, including the size and duration of OC lesions, act together to influence the sensitivity of different relaxation constants to PG content. Interestingly, extensive PG loss (5, 9, 10 weeks post surgery) was always associated with dramatic increases in T_1ρ_ and adiabatic T_1ρ_ relaxation times. Conversely, when there was no cartilage necrosis and the PG content was only slightly reduced (6 weeks post surgery), T_1ρ_ and adiabatic T_1ρ_ increased only up to 30% in the operated area, less than T_2_ and T_RAFF_. These results suggest that T_1ρ_ and adiabatic T_1ρ_ correlate better to changes in PG content between viable vs. necrotic cartilage than T_2_ and T_RAFF_.

The most important results of our study are the high sensitivity of almost all MR parameters (with the exception of T_1_) to detect and delineate lesions of cartilage necrosis, along with the finding of similar sensitivity of relaxation parameters in the laboratory frame (T_2_), the first rotating (T_1ρ_ and adiabatic T_1ρ_) and the second rotating (T_RAFF_) frames. PG synthesis is likely immediately interrupted after the surgical procedure used to induce cartilage necrosis, but the existing naturally high proteoglycan [8] content is decreasing slowly over time. It has been confirmed as early as 1985 that PG-content in OCD lesions is extremely low [8] when compared to normal epiphyseal cartilage. As reflected in our results, there is a graded response to injury, where the degree of matrix compositional changes in the smaller and more acute lesions is much less than in the larger and/or more chronic lesions. At 6 weeks after induction of a small lesion, H&E-staining did not detect changes consistent with chondronecrosis, but the parametric MRI method was sensitive to the subtle changes that were also detected by the optical density measurements (Figs 1E and 2E).

The entire process of enchondral ossification during development consists of replacement of epiphyseal cartilage with bone. In the case demonstrated in Fig 1F, the original lesion of epiphyseal cartilage necrosis was completely surrounded (not replaced) by the advancing ossification front, therefore became inconsequential for normal function and could be considered “healed”. Therefore, the quantitative assessment of osseous changes also deserves attention, but is beyond the scope of this paper.

For traditional parametric MRI methods used for assessment of necrotic cartilage, such as T_2_ and T_1ρ_, our results at high field (9.4T) are consistent with the reports at low fields used for clinical scanners (1.5T and 3T) [16]. Since adiabatic T_1ρ_ and T_RAFF_ have not been implemented in clinical diagnosis of OCD at 1.5T or 3T, systematic comparisons between the high field and low field should be conducted for adiabatic T_1ρ_ and T_RAFF_ in future studies.

For the viable epiphyseal cartilage, the relaxation times fluctuated at different time points. During maturation of the epiphysis, biochemical changes in, for example, collagen and PG contents could influence the relaxation constants. In addition, the estimations of the relaxation constants were conducted based on 2D MRI instead of 3D MRI, thus the deviation could also arise due to not utilizing the entire volume of the epiphyseal cartilage for estimation. Further investigations of the healthy epiphyseal cartilage should be conducted in future studies, but are beyond the scope of this work.

There are limitations to our feasibility study, including the relatively small number of animals that underwent the surgical procedure and subsequent measurements up to 10 weeks following the procedure. Also, when considering human applications, the SAR may become a concern. Compared to conventional T_2_ mapping using multiple echo times, T_1ρ_ and adiabatic T_1ρ_ continuously deposit RF energy into the patient during the magnetization preparation, which results in a risk of high SAR. Without any acceleration techniques utilized in the image acquisition, considerable time was required to measure all the different relaxation times. In the future, acceleration techniques, such as parallel imaging and multiband excitation, could be utilized to shorten the scan time for clinical applications [39–42].

## Conclusions

In this work, a comprehensive evaluation of multiple parametric MRI methods has demonstrated a high sensitivity of T_2_, T_1ρ_, adiabatic T_1ρ_ and T_RAFF_ for detecting and following focal lesions of chondronecrosis of variable duration in epiphyseal cartilage in a goat model of OC. This suggests that parametric MRI may be able to provide information about the size and duration of OC lesions. The decision regarding which parametric measures are selected should be governed by the requirements of a specific application. Since the native T_2_ relaxation proved to be as sensitive as the higher rotating frame methods, MRI applications using vendor provided sequences and post-processing packages are highly attractive for future studies on juvenile OCD in the clinical setting.
